# Submerged Vegetation and Water Quality Degeneration From Serious Flooding in Liangzi Lake, China

**DOI:** 10.3389/fpls.2019.01504

**Published:** 2019-11-25

**Authors:** Ligong Wang, Yuqin Han, Haihao Yu, Shufeng Fan, Chunhua Liu

**Affiliations:** The National Field Station of Freshwater Ecosystem of Liangzi Lake, College of Life Science, Wuhan University, Wuhan, China

**Keywords:** flooding, submerged vegetation, dominant species, water quality, diversity

## Abstract

In shallow lake ecosystems, flooding is a key disturbance factor of aquatic vegetation. Aquatic plants, especially submerged plants, play key roles in water ecosystems. Liangzi Lake experienced severe flooding in July 2010, and the elevated water levels lasted for 3 months. In this study, 10 transects with 120 monitoring points were set up for monthly monitoring during the 3-year period, encompassing the period before and after the flooding (2009–2011). The numbers, biomass, and diversity of the submerged plants, as well as the physical and chemical characteristics of the lake water, were surveyed. There were 12 species belonging to 7 families and 7 genera in Liangzi Lake. Eleven of the submerged plant species were found in 2009, but, after the flood, that number decreased to five in 2011. The total biomass differed significantly over the three years (*P* < 0.05), with the largest biomass in 2009 and smallest in 2011. In 2009 and 2010, *Potamogeton maackianus* was the dominant species, but its dominant position weakened in 2011. After the flood, water transparency decreased, and the water depth, turbidity, total nitrogen, and total phosphorus increased. A redundancy analysis between the submerged plants and environmental factors found that the water transparency, turbidity, and water depth were the key environmental factors affecting the plants. These results suggest that the long-lasting severe flooding of Liangzi Lake in 2010 led to the degradation of both the submerged plant community and water quality.

## Introduction

Submerged macrophyte vegetation plays a central role in the functioning of shallow lake ecosystems (Coops and Doef, 1996; Jeppesen et al., 1997; Meerhoff et al., 2003). It can provide feeding and spawning habitats for fish, provide sanctuary for zooplankton, and generally help improve the biodiversity and stability of lake ecosystems (Jeppesen et al., 1998; Wetzel, 2001; Heikkinen et al., 2009; Tamire and Mengistou, 2013; Yu et al., 2016). Among lakes, the factors influencing the submerged macrophyte distribution, diversity, and abundance include light availability (Middelboe and Markager, 1997; Phillips et al., 2016; Zhang et al., 2016; Verhofstad et al., 2017), water temperature (Scheffer et al., 1992; Short et al., 2016), nutrient enrichment (Sand‐Jensen et al., 2008), bottom substrate (Andersson, 2001), herbivory (Marklund et al., 2002; Sponberg and Lodge, 2005), and the water level (Wilcox and Meeker, 1991). In shallow lake ecosystems, water level fluctuations are the main factor affecting the biomass and spatial distribution of aquatic plants and are an important ecological factor affecting their growth and reproduction (Gafny and Gasith, 1999; Strand and Weisner, 2001; Ishii and Kadono, 2004; Deegan et al., 2007; Schneider et al., 2018).

Flood is one of the important factors leading to fluctuation of water levels (Wantzen et al., 2008). In addition to a rise in the water level, the surface runoff caused by floods carries large amounts of potentially labile nitrogen and phosphorus into the lake, and the original endogenous nutrients used by aquatic plants are also released into the water (Carpenter, 2008; Keitel et al., 2016). Floods also resuspend the sediment, increasing the concentrations of suspended solid particles, nitrogen, and phosphorus in the water (Newman and Reddy, 1992; Tong et al., 2017). The phosphorus released by the resuspension of precipitates is 20–30 times higher than when they are undisturbed (Søndergaard et al., 1992). In addition, floods also restrict the availability of oxygen, inhibiting the growth of emergent and floating-leaved plants (Drew, 1997; Deegan et al., 2007; Lemke et al., 2014) and the germination of some species in the seed bank (Casanova and Brock, 2000; Johansson and Nilsson, 2002; Hölzel and Otte, 2004; Cui et al., 2017), thereby reducing the diversity of aquatic plant species (Jeppesen et al., 2015). Correspondingly, aquatic animal habitat and food sources also disappear, reducing species diversity, and thus the entire ecosystem becomes very vulnerable (Junk and Robertson, 1997; Dorn and Cook, 2015). In addition, climate change manifested through increasing temperatures and more variable precipitations impacted water quality, biodiversity, and ecological status of the world’s lakes (Solheim et al., 2010; O’Reilly et al., 2015). Climate change is predicted to lead to earlier, stronger, and more frequent flooding (Fowler and Hennessy, 1995; Trenberth, 2011; Cai et al., 2015; Lehmann et al., 2015). For example, floods have become more frequent in the central United States (Hirsch and Archfield, 2015), and global warming has been linked to a substantial increase in flood risk in most countries in Central and Western Europe (Alfieri et al., 2018). The high frequency of future floods may have a more serious impact on water ecosystems (Watts et al., 2015; Castello and Macedo, 2016).

Flooding is a key disturbance factor of aquatic vegetation composition and community diversity in floodplain lakes (Tockner et al., 2000; Maltchik et al., 2005; Van Geest et al., 2005; Chaparro et al., 2014). The growth of emergent floating-leaved plants is not limited by low light penetration in the lake (Qiu et al., 2001a). Spate floods affected small to intermediate-sized submerged plant species, and long-term inundating floods affected tall submerged plant species (Bornette and Puijalon, 2011). The effects of water levels on submerged plants by simulating water level fluctuations for individual plants have been studied in depth (Armstrong et al., 1994; Vartapetian and Jackson, 1997; Vermaat et al., 2000; Lenssen et al., 2004; Wang et al., 2016a). For example, water depths greater than 3 meters severely reduced the survival of *Vallisneria natans* (Han et al., 2018). *Potamogeton maackianus* disappeared at an average depth of 6 meters in Erhai Lake (Fu et al., 2018a). *Myriophyllum spicatum*, *Ceratophyllum demersum*, and *Potamogeton malaianus* were more tolerant of deep water and flood intensity than *P.maackianus* and *Hydrilla verticillata*, as indicated by their larger biomass, plant height, stem tensile properties, and root anchorage strength (Zhu et al., 2012; Ye et al., 2018). The response of submerged plants to floods is species specific. Therefore, flooding with extreme water levels may cause shifts towards a macrophyte-dominated state (Coops et al., 2003).

In recent decades, floods have become more frequent in the middle reaches of the Yangtze River in China, and the rise in water levels has been greater than before (Li et al., 2015; Wang and Yuan, 2018). Lake Liangzi is located in the middle and lower reaches of the Yangtze River. From 2007 to 2016, two major floods occurred in Lake Liangzi, one in 2010 and one in 2016 (Xu et al., 2018). Ten transects with 120 monitoring points were set up for monthly monitoring during the 3-year period between 2009 and 2011 in order to compare the submerged vegetation and water characters before and after the flood in 2010. Specifically, we analyzed the relationship between submerged aquatic communities and water quality. Finally, we evaluate the consequences of flood regulation on the dominant submerged species.

## Materials and Methods

### Study Area and Flood

Liangzi Lake (30°04′55″–30°20′26″ N, 114°31′19″–114°42′52″ E) is located south of the Yangtze River in the southeast of Hubei Province, China. It is a typical grass-type lake, a type common in East China, with high vegetation coverage. The lake covers an area of 304.3 km^2^ and has a water storage capacity of about 14 × 10^8^ tons.

**Figure 1:**
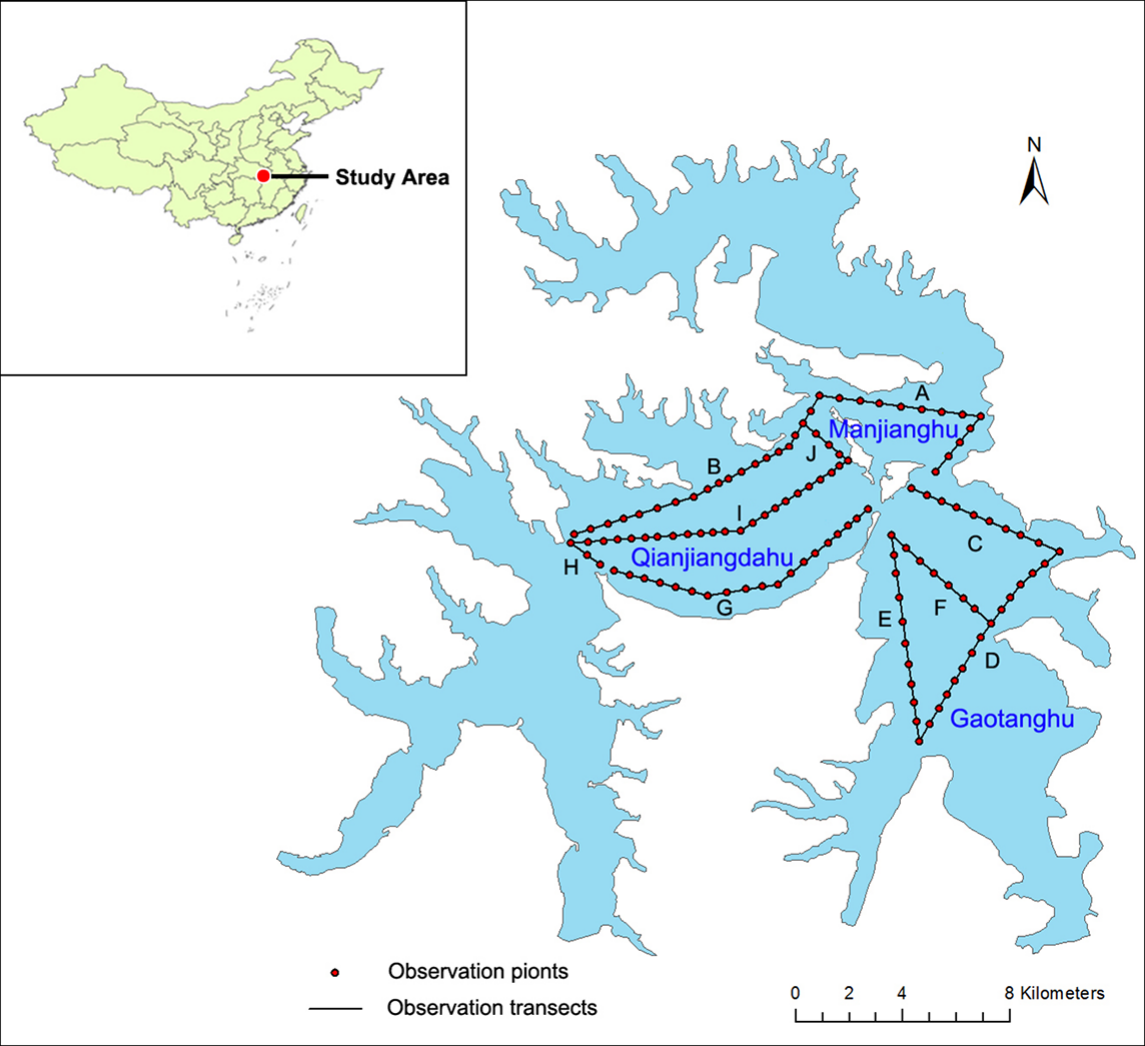
Map of Liangzi lake showing sampling sites observation points. Ten transects: A. B. C. D. E. F. G. H. I. J.

The Liangzi Lake Basin suffered heavy rain, and Liangzi Lake was seriously flooded in July 2010, with the water level rapidly rising from 4.25m to 6.2m. In addition, the high water levels continued for three months. The survey was conducted for monthly monitoring from January 2009 to December 2011. Monitoring plots were established in three regions of Liangzi Lake (named as Qianjiangdahu Lake, Manjianghu Lake, and Gaotanghu Lake) (**Figure 1**). Altogether, there were ten transects, with 120 monitoring points for the sampling set up at 400-meter intervals (**Figure 1**).

### Collection of Plant Samples and Water Parameter Measurements

During each survey, submerged plant samples were collected on-site using a boat positioned by GPS (GARMIN eTrex Summit; Garmin, Inc., Olathe, KS, USA) navigation. At each monitoring site, the submerged plants were randomly sampled twice with a Peterson’s mud filter (0.2m × 0.3m). The collected samples were packed in plastic bags (0.03 L) and brought to the National Field Station for Freshwater Ecosystem at Liangzi Lake (hereinafter referred to as the Liangzi Lake National Station). The plants from the monitoring sites were first classified, the number of each species was counted, and they were then drained of surface water and weighed using an electronic scale (0.01g) to obtain the wet weight. Some plant samples were dried at 80°C for 72 h and were then weighed to obtain dry-weight biomass, which was later converted to a submerged plant dry weight per unit area (1m^2^).

Water parameters were monitored at the time of the submerged plant sampling. The water pH, dissolved oxygen, and temperature were measured using a Pro Plus water quality monitor (YSI Inc., Yellow Springs, OH, USA); the water turbidity was measured in nephelometric turbidity units (NTUs) using a HACH 2100P turbidity meter (HACH Co., Loveland, CO, USA); and the water depth and transparency were measured by Secchi depth monitoring. A Plexiglass water sampler was used to collect water samples; ten sites were sampled once a month from 2009 to 2011, and these were returned to the instrument room of the Liangzi Lake National Station where the total phosphorus and total nitrogen were determined with HACH IL500P and IL500N analyzers (HACH Co., USA). We thus had a total of 4,320 samples (3 years × 12 months × 120 sites), 360 total phosphorus, and total nitrogen samples (3 years × 12 months × 10 sites).

### Data Analysis

The submerged plant diversity index was analyzed using the Shannon index formula:

$$\text{H}=-\sum (\mathrm{Pi})(\log_{2}\mathrm{Pi})\;(\text{MaGuarran},1988).$$

Only six submerged species were common in Liangiz Lake. Thus, the dominance analysis and redundancy analysis (RDA) were analyzed with the data for these six species. The dominance of the submerged plant species was calculated with the equation:

Dominance =[(relative frequency + relative weight)/2]×100% (Chen,1980).

The data analyses were conducted using SPSS 22.0 software. To ensure that all data met the normal distribution requirements, data that were not normally distributed underwent a logarithmic transformation, but, to the data that were not normally distributed, non-parametric statistics were applied. We conducted a Kruskal–Wallis test to determine the differences in the water quality. A one-way ANOVA with Duncan’s (*P* < 0.05) test for *post hoc* comparison was used to analyze the differences in species number, total biomass per area, total biomass of dominant species among 2009, 2010, and 2011, or during the same month over different years. A redundancy analysis (RDA) based on the biomass was conducted for the major water environmental factors affecting the submerged plant communities using Canoco for Windows 5.0 software.

## Results

### Species Number, Total Biomass and Diversity Index

**Figure 2:**
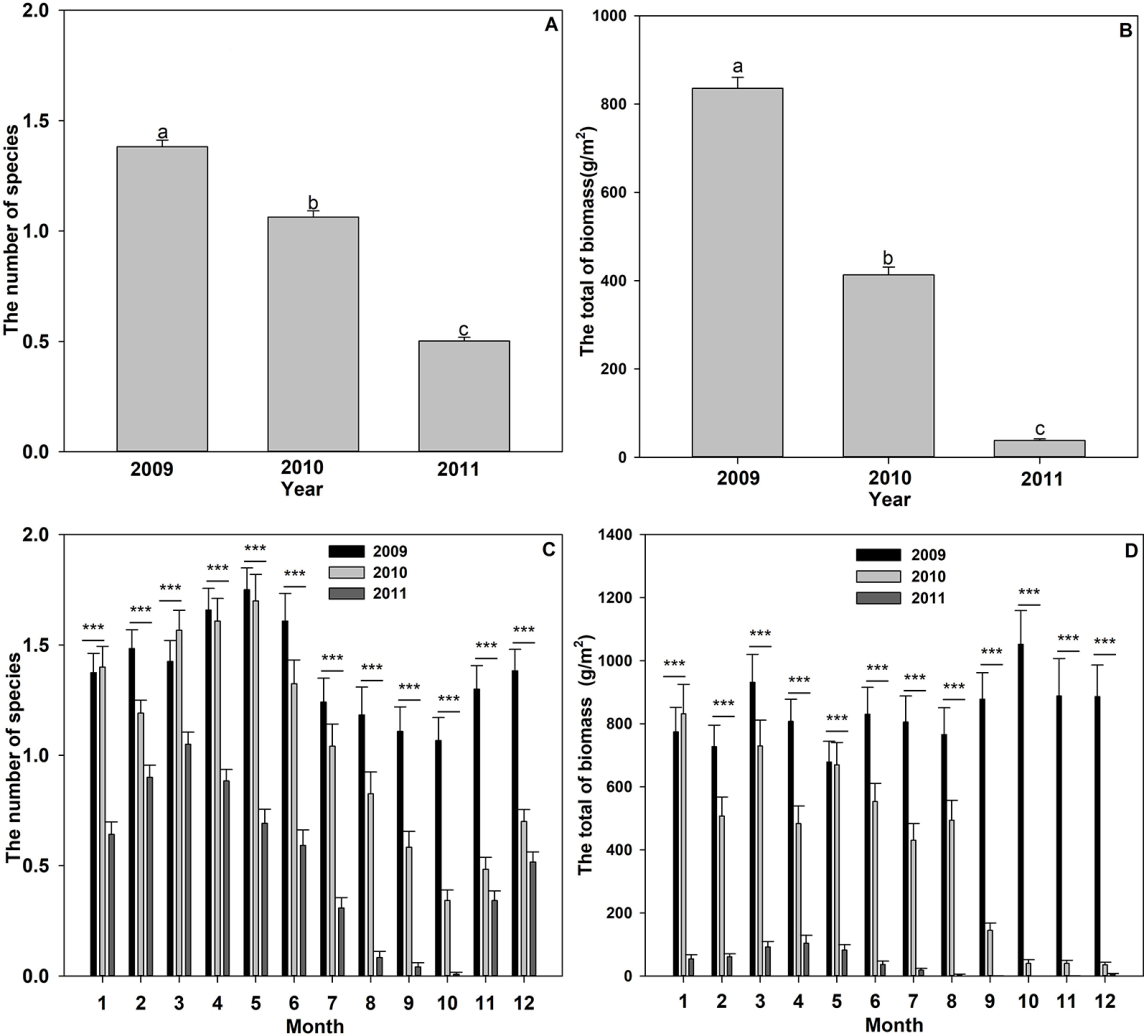
Submerged species number **(A**, **C)** and the total biomass **(B**, **D)** in different years or different months. Values shown are means ± S.E. Bars with different lowercase letters above are significantly different. Significant differences: ***P < 0.001.

Twelve submerged plant species were monitored in Liangzi Lake from 2009 to 2011, which belonged to seven genera in seven families (**Table 1**). There was a significant change in the number of submerged plant species over the three years (**Figure 2A**), the number of species in 2009 being significantly higher than that in 2010 and 2011 (**Figure 2A**). A comparison of the number of species before and after flooding found no significant differences from March to July in 2009 and 2010, while the number present from August to December in 2009 was significantly higher than that in 2010 (**Figure 2C**). There were significant differences in the number of submerged plants with each month within the three years (**Figure 2C**).

**Table 1:** Submerged plant species in Lake Liangzi.

Family	Species
Characeae	*Chara vulgaris*
Ceratophyllaceae	*Ceratophyllum demersum*
Haloragaceae	*M. spicatum*
Hydrocharitaceae	*H. verticillata*
	*Elodea nuttallii*
	*Vallisneria spiralis*
Lentibulariaceae	*Utricularia aurea*
Najadaceae	*Najas marina*
	*N.minor*
Potamogetonaceae	*Potamogeton crispus*
	*P. maackianus A. Bennett*
	*P.malaianus*

There were significant differences in total biomass over the three years(*F* = 504.227, *P* < 0.001, the largest biomass identified in 2009 and the smallest in 2011 (**Figures 2B, D**). According to monthly data, the three years also had significant differences in the total biomass during each month (All *P* < 0.05). The total biomass of the submerged plants differed significantly from each other in February, April, June, July, and August (**Figure 2D**). In January, March, and May, the total biomass of the submerged plants in 2009 showed no significant difference to that in 2010, whereas both them were significantly higher than in 2011 (**Figure 2D**).

**Figure 3:**
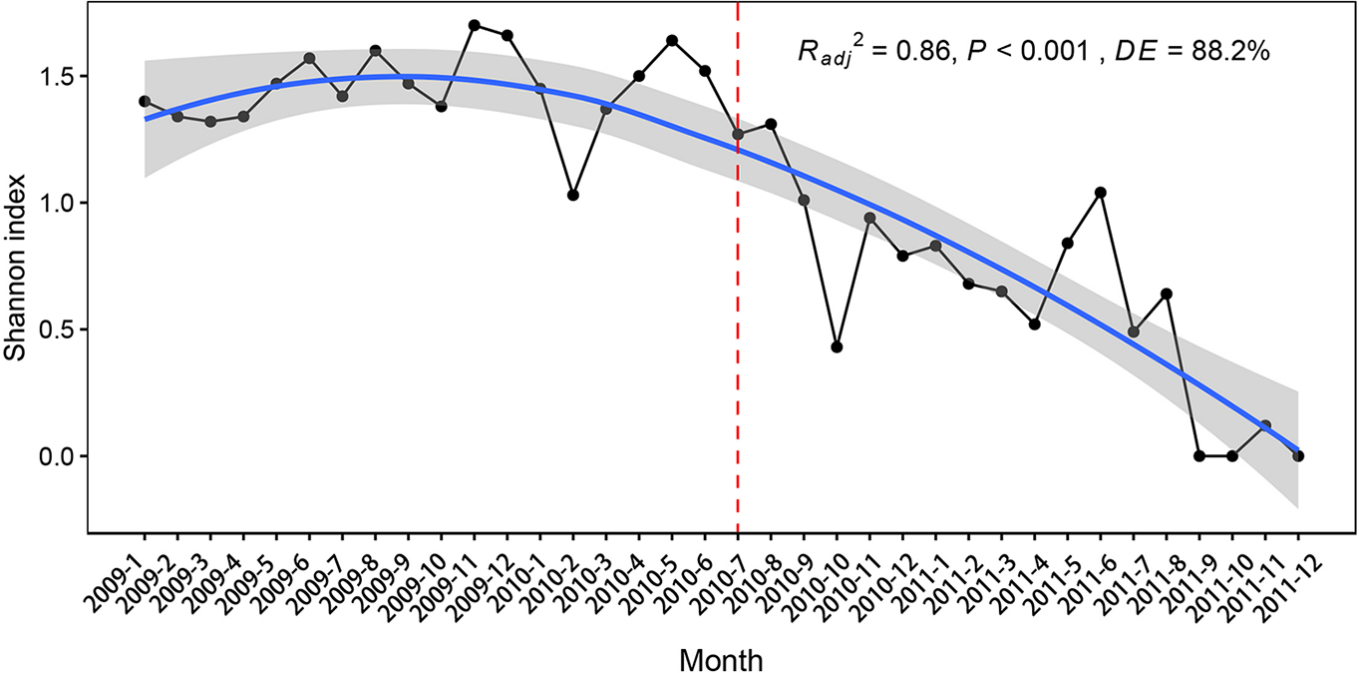
The Shannon diversity species index of the submerged plants in 2009, 2010, and 2011.

Flooding decreased the Shannon diversity index (**Figure 3**). The Shannon index was highest in November 2009, while only one species was found in September, October, and December 2011, resulting in the lowest diversity index (**Figure 3**).

### Changes in the Dominant Species

**Figure 4:**
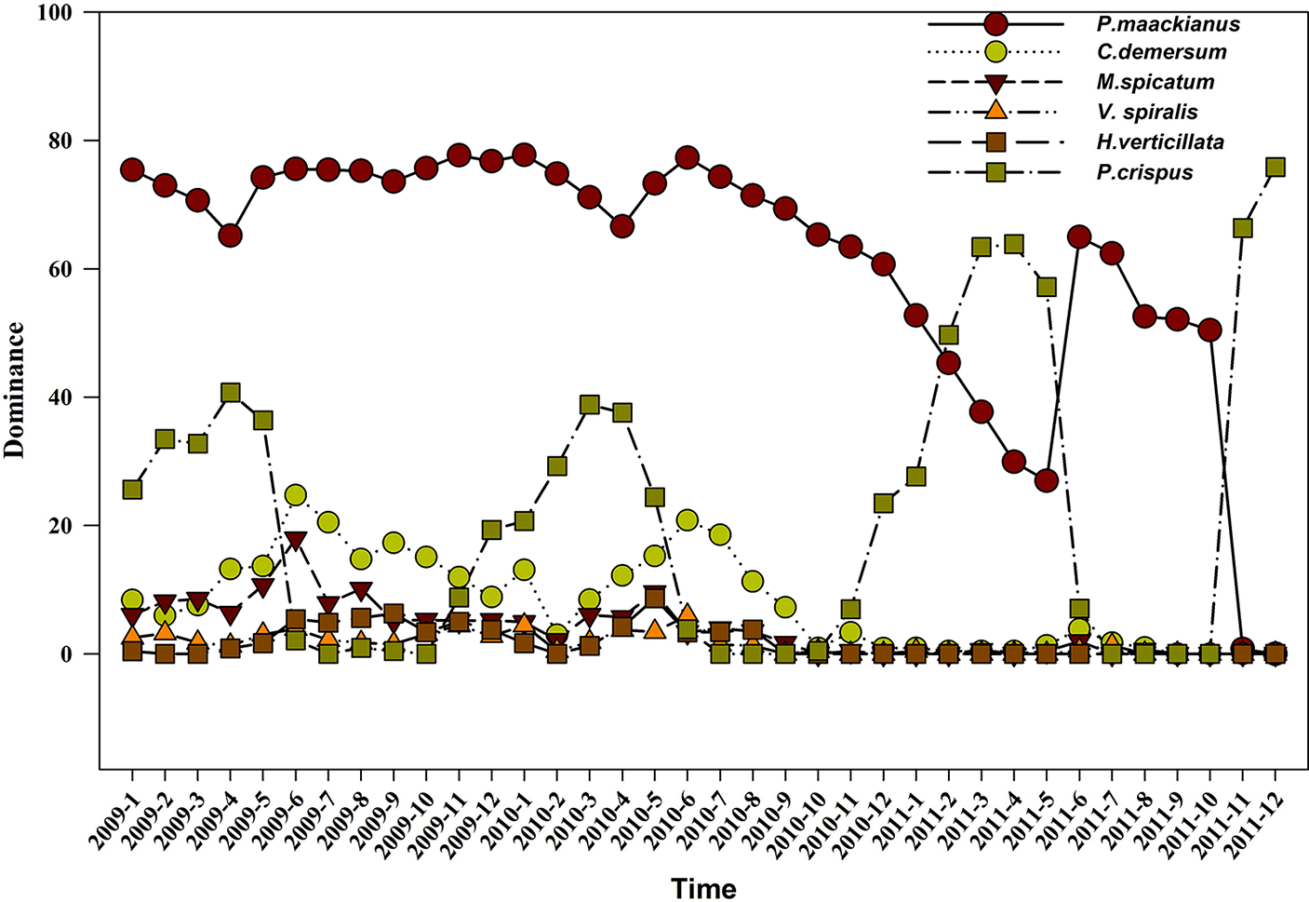
The species dominance of the submerged plants in 2009, 2010, and 2011.

In the three years sampled, the dominance of *P. maackianus* was above 60%, while the dominance was less significant among *C. demersum*, *M. spicatum*, and *P. crispus*. *P. maackianus* was the dominant species during whole year both in 2009 and 2010. However, the dominant species was *P. crispus* in March, April, May, November, and December of 2011, and only *P. maackianus* was present from June to October 2011 (**Figure 4**).

### Water Environmental Parameters

There were significant difference in water depth (χ**^2^** = 871.013, *P* < 0.001), transparency (χ**^2^** = 1667.673, *P* < 0.001), turbidity (χ**^2^** = 1649.164, *P* < 0.001), dissolved oxygen (χ**^2^** = 218.637, *P* < 0.001), pH(χ**^2^** = 804.817, *P* < 0.001), total nitrogen (χ**^2^** = 1165.63, *P* < 0.001), and total phosphorus (χ**^2^** = 1704.382 *P* < 0.001) over the three years, There was no significant difference in temperature (χ**^2^** = 2.4722, *P* = 0.291).

**Figure 5:**
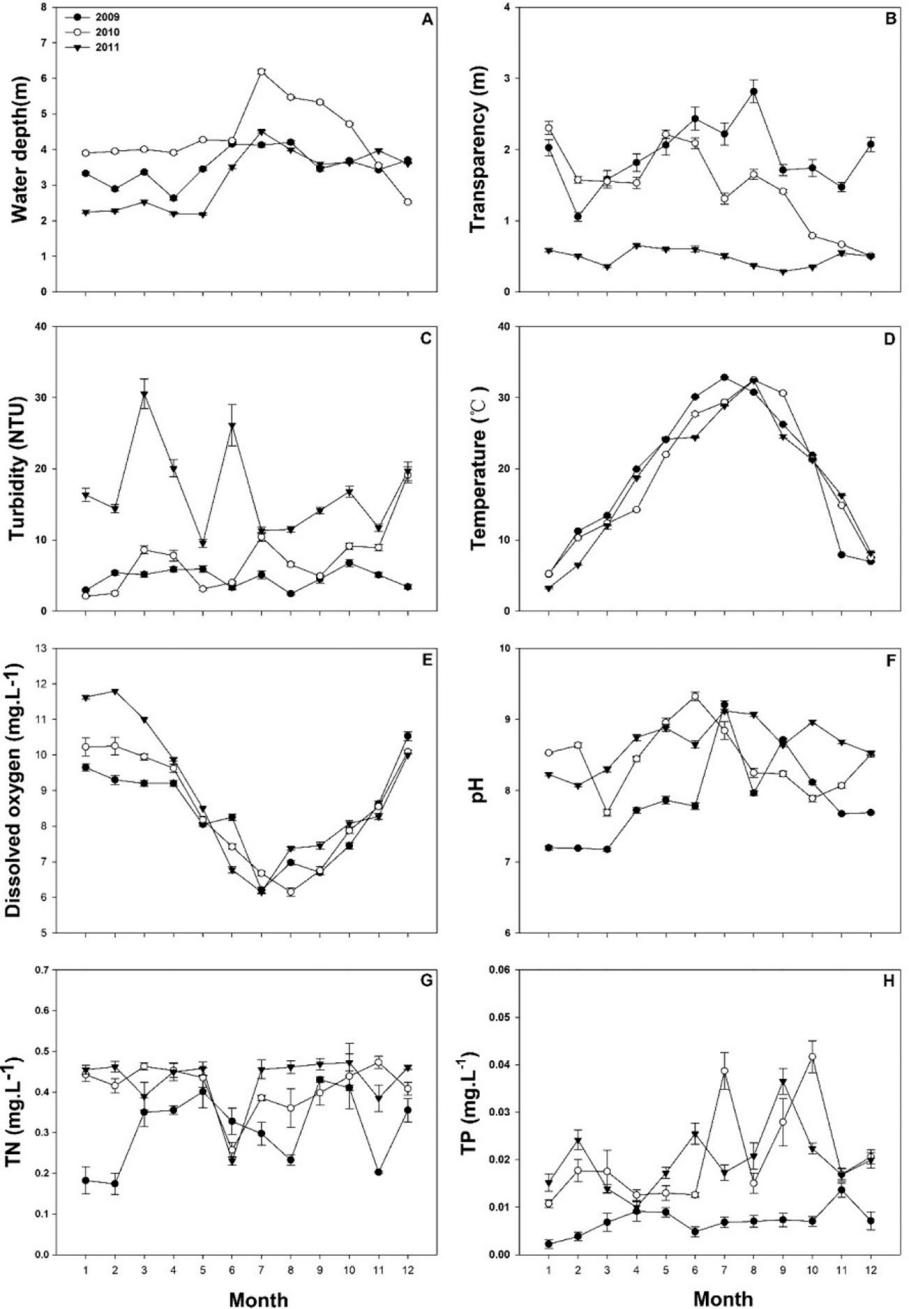
The water parameters of Lake Liangzi. **(A)** water depth, **(B)** transparency, **(C)** turbidity, **(D)** temperature, **(E)** dissolved oxygen, **(F)** pH, **(G)** total nitrogen (TN), **(H)** total phosphorus (TP).

The mean water depth of Lake Liangzi from January to October was greater in 2010 than in 2009 and 2011 (**Figure 5A**). On the other hand, water transparency was significantly lower in 2011 than that in 2009 and 2010, and the maximum transparency was found in August 2009 (**Figure 5B**). Water turbidity was significantly higher in 2011 than that in 2009 and 2010, reaching its highest value in March 2011 (**Figure 5C**). The highest water temperature values were reached in August, with an average maximum temperature of 34°C, and lowest values were present in January, when the average minimum temperature was 3°C (**Figure 5D**). In contrast to the temperature, dissolved oxygen had an inverse trend, decreasing in the summer and increasing in the winter (**Figure 5E**). The lowest dissolved oxygen concentration values were present in July, with a mean of 6.34 ± 0.793mg·L^-1^ (**Figure 5E**). The pH was significantly higher in 2011 than in 2009 (**Figure 5F**). Total nitrogen (TN) was lower in 2009 (0.310 ± 0.01mg·L^-1^) than in 2010 (0.411 ± 0.011mg·L^-1^) and 2011(0.429 ± 0.109 mg·L^-1^). (**Figure 5G**). Total phosphorus (TP) fluctuated slightly in 2009, whereas it fluctuated widely in 2010 and 2011 (**Figure 5H**). The mean value was 0.007 ± 0.0005mg·L^-1^in 2009, and it was significantly less than in 2010 (0.020 ± 0.001 mg·L^-1^) and 2011 (0.020 ± 0.0008 mg·L^-1^) (**Figure 5H**).

### Redundancy Analysis (RDA) of Submerged Macrophyte Communities and Water Environmental Cactors

The eight environmental factors cumulatively accounted for 41.17% of the species change information in the two axes. A Monte Carlo displacement test showed that the eight environmental factors were significant (*P* = 0.002), indicating that the transparency (which explained 36.8% of the variability with a correlation of 72.05% with the presence of submerged macrophytes), turbidity (which explained 11.9% of the variability), and water depth (which explained 8.3% of the variability) were factors affecting the structure of the submerged plant communities and were, therefore, key environmental water factors.

**Figure 6:**
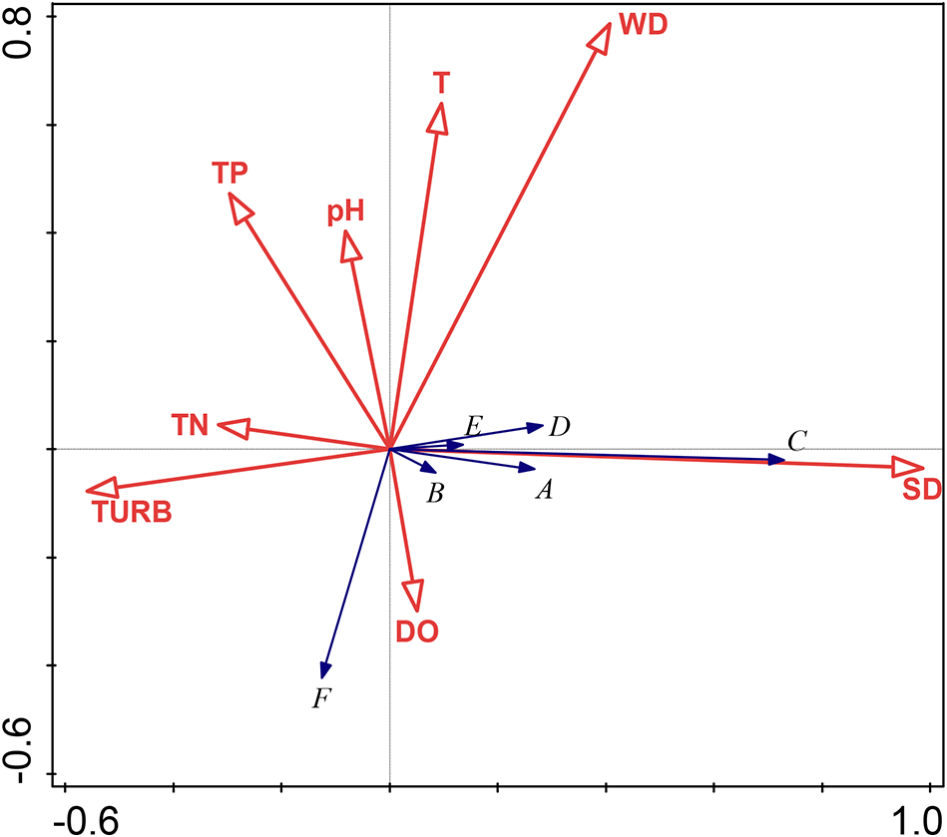
Redundancy analysis ordination diagram of the submerged plant and environmental factors. Species codes: A, *Myriophyllum spicatum*; B, *Vallisneria spiralis*; C, *Potamogeton maackianus A. Bennett*; D, *Ceratophyllum demersum*; E, *Hydrilla verticillata*; F, *Potamogeton crispus*. Environmental codes: DO, Dissolved oxygen; pH, pH of water; SD; Transparency; T, Temperature; TN, Total nitrogen; TP, Total phosphorus; TURB, Turbidity; WD, Water depth.

The gradient of the first axis from left to right shows that as the transparency increases and turbidity decreases, and the submerged macrophytes (except *P. crispus*) are distributed in the areas of high transparency (i.e., the positive direction of the first axis) (**Figure 6**). *P. maackianus* was related to the first axis and significantly positively correlated with transparency (*P* < 0.001); dissolved oxygen is significantly related to the second axis and negatively correlated with temperature (*P* < 0.001) (**Figure 6**).

## Discussion

### Dynamic Changes in the Submerged Plant Communities

In shallow lake ecosystems, the water level is the main factor affecting aquatic plant biomass (Wallsten and Forsgren, 1989; Zhang et al., 2016), and the natural water level is a dynamic factor (Rea and Ganf, 1994). High water levels caused by extreme flooding are known to reduce the diversity of aquatic plant species (Jackson, 1984; Arias et al., 2018). For example, flooding increased the water level and decreased diversity and biomass of the aquatic vegetation; the most serious effect observed was in submerged plants in Poyang Lake in China (Cui et al., 2000). In our survey, after the flood in 2010, the total number of species in Lake Liangzi decreased from ten species in 2009 to five in 2011, and the average number of species per site decreased from 1.38 ± 0.03 per m^2^ in 2009 to 0.5 ± 0.017 per m^2^ in 2011. The diversity and biomass of submerged plants were all significantly decreased by flooding in 2010. Those decreases were mainly due to a medium-term 3 m rise in the water level within several days, and this reached more than 6 m for a brief time; consequently, such an increased water depth significantly inhibited the growth of many submerged plants (Wang et al., 2016b). In addition, there was a negative relationship between submerged macrophyte dominance and the long-term annual duration of inundation (Van Geest et al., 2003). Thus, the long duration of higher water levels (a greater than 4 m increase in water level that persisted for four months from July to October) caused by the flooding of Lake Liangzi in 2010 resulted in the dying-off of a large number of the submerged plants, and the dry biomass thus decreased significantly. On the other hand, a certain period of time is required for plants to adapt to different water levels (Bornette and Puijalon, 2011). The lack of significant differences in the number of species in July and August of 2009 and 2010 suggests that the submerged plants in Lake Liangzi had some tolerance for the short-term changes in water levels during flooding.

The RDA analysis showed that water transparency, turbidity, and water depth were the key water environmental factors affecting the submerged plants (**Figure 6**). The increase in water level leads to reduced light availability in shallow lakes, thereby limiting the growth of submerged plants (Van Geest et al., 2007). Decreasing water transparency significantly decreased the communities in terms of biomass, and it also decreased the submerged plants species’ richness (Vestergaard and Sand-Jensen, 2000; Wang et al., 2016b). The key factor determining whether submerged plants can regenerate is the underwater light conditions during the germination period of the plants’ vegetative propagules (Lu et al., 2012). Weak underwater light intensity prevents germination, thus the number of species decreases (Madsen et al., 2001). Thus, when sediment is disturbed by flooding, it causes the water transparency to decrease and the turbidity to increase, and this lack of underwater light affects the growth and reproduction of the submerged plants. In the present study, the water turbidity increased, the transparency of the water decreased after flooding, and these factors inhibited the growth and regeneration of submerged plants.

*P. maackianus* is the dominant species in the submerged vegetation of many lakes in the middle and lower reaches of the Yangtze River (Li et al., 2004). It is a constructive species in submerged plant communities, and the distribution area of the *P. maackianus* community once accounted for 50% of the total area of submerged plants in Lake Liangzi (Zhan et al., 2001). We also found that it was the dominant species in Lake Liangzi (dominance > 60%) in 2009 and 2010. However, after the 2010 flooding, the dominant species was *P. crispus* in February, March, April, May, November, and December of 2011. P. *maackianus* was the dominant species only from June to October of 2011 (**Figure 4**), which was mainly because the summer buds (dormant buds) of *P. crispus* germinate in the autumn and then grow over the winter. It was thus able to become the dominant species from February to May in 2011. Although *P. maackianus* can grow in winter, the flooding caused turbidity to increase, water transparency to decrease, and light intensity to weaken, resulting in the *P. maackianus* gradually dying. The tolerance of the summer buds (dormant buds) of *P*. crispus is strong. For example, higher water turbidity (90NTU) had no effect on the germination rate and growth of summer buds (Li, 2012), whereas four meters of water depth significantly affected the growth of *P. maackianus* (Zhu et al., 2012; Li et al., 2013), In addition, previous studies have found that *P. crispus* can successfully recover, while it has been difficult to successfully restore *P. maackianus* because *P. maackianus* are K-selected plants (Qiu et al., 2001b; Zhu et al., 2012; Fu et al., 2018b) and *P*. crispus are r-selected plants (Pierce et al., 2012).

### Dynamic Changes in the Water Environmental Factors

The submerged macrophytes improve their own light climate by enhancing the water transparency (Van den Berg et al., 1998). There is a significant positive relationship between water transparency and the maximum colonization depth of aquatic plants (Canfield et al., 1985; Sondergaard et al., 2013). These two parameters of the water before the flooding in 2009 were stable due to the high species numbers and biomass of submerged vegetation. Floods have an important effect on water clarity (Xu et al., 2018), and extreme water levels may cause shifts between the turbid and the clear, and the macrophyte-dominated state may change to a without-vegetation turbid state (Coops et al., 2003; Scheffer and Carpenter, 2003). The flooding of Lake Liangzi in 2010 caused the turbidity to increase and, consequently, the water transparency to decrease. In addition, large areas of aquatic vegetation disappeared in Lake Liangzi after the flood. Submerged plants were only found at five monitoring points and one monitoring point in September and October 2011.

Nutrient input, mainly of N and P, is derived from the eutrophic main channels during floods (Van den Brink et al., 1994). A large amount of suspended sediment and, consequently, a higher concentration of nutrients into Lake Liangzi is caused by flooding that increases the content of nitrogen and phosphorus in water. During the growth phase, the water column is depleted in nutrient concentrations, whereas, during the decay period, there is a significant increase in water column nutrients (Shilla et al., 2006). Furthermore, the decomposition of submerged macrophytes is influenced by several factors, though water temperature has been cited as an important environmental factor (Carpenter and Adams, 1979; Carvalho et al., 2005). After the flooding in Lake Liangzi it was still a hot season, the higher temperature accelerating the decomposition of dead aquatic plants caused by the flood in 2010. Higher turbidity, higher total nitrogen and phosphorus, and lower transparency after the flooding in 2010 all contributed to the downward trend in water quality.

## Conclusion

The serious flooding of 2010 in Lake Liangzi decreased species diversity and the biomass of submerged aquatic plants and resulted in declining water quality. *P. maackianus* was the dominant submerged species during the whole year before flooding, while this dominant position weakened after the flooding. The results suggest that heavy flooding may change the submerged community succession.

## Data Availability Statement

All datasets generated for this study are included in the manuscript and the supplementary files.

## Author Contributions

CL designed the study. LW, YH, and SF performed the field monitoring. LW, YH and HY analyzed the data. LW drafted the manuscript with the assistance of CL. All the co-authors commented on and approved the final manuscript.

## Conflict of Interest

The authors declare that the research was conducted in the absence of any commercial or financial relationships that could be construed as a potential conflict of interest.

The handling editor is currently organizing a Research Topic with one of the authors CL, and confirms the absence of any other collaboration.
